# Major Earthquakes of the Past Decade (2000-2010): A Comparative Review of Various Aspects of Management

**DOI:** 10.5812/traumamon.4519

**Published:** 2012-05-26

**Authors:** Mohammad Hosein Kalantar Motamedi, Masoud Sagafinia, Ali Ebrahimi, Ehsan Shams, Mostafa Kalantar Motamedi

**Affiliations:** 1 Trauma Research Center, Baqiyatallah University of Medical Sciences, Tehran, IR Iran

**Keywords:** Earthquakes, Review, Disasters, Risk Management

## Abstract

**Objectives:**

This article sought to review and compare data of major earthquakes of the past decade and their aftermath in order to compare the magnitude, death toll, type of injuries, management procedures, extent of destruction and effectiveness of relief efforts.

**Materials and Methods:**

A retrospective study of the various aspects of management and aftermath of 5 major earthquakes of the past decade (2000–2010) was undertaken. This included earthquakes occurring in Bam Iran, Sichuan China, Port-au-Prince Haiti, Kashmir Pakistan and Ica Peru. A literature search was done via computer of published articles (indexed in Pubmed). The issues assessed included: 1)Local magnitude,2)Type of building structure 3)Time of the earthquake (day/time/season), 4)Time to rescue, 5)Triage, Transfer, and Treatment 6) Distribution of casualties (dead/ injured), 7)Degree of city damage, 8)Degree of damage to health facilities, 9)Field hospital availability, 10)International aid, 11)Air transfer, 12) Telecommunication systems availability, 13) PTSD prevalence, 14) Most common injury and 15) Most common disease outbreak.

**Results:**

The Bam earthquake had the lowest (6.6 Richter’s) and the Sichuan earthquake had the greatest magnitude (8.0 Richter’s). Mortality in Haiti was 212,000 and it was the deadliest earthquake of the past decade. Collapse of heavy clay roofing structures was a major cause of death in Iran and Pakistan. Earthquakes occurring at night and nonworking days carried a high death toll. The time to rescue and treat was the lengthiest in Haiti (possibly contributing to the death to injured ratio). However, the worst dead to injured ratios were in Bam (51%) and in Pakistan (47%); the best ratio was in China (15%). Iran and Pakistan suffered the highest percentage of damage to the health facilities (90%). Field hospital availability, international aid and air transfer were important issues. Telecommunication systems were best in China and worst in Pakistan. PTSD prevalence was highest in Iran. Respiratory infection was the most common infection following all 5 earthquakes.

**Conclusions:**

Earthquake damage, death toll, managerial protocols etc. vary in different countries and are influenced by many factors including the hour the earthquake hits and the day of the week. Additionally, social, structural and geographic factors as well as the medical, governmental and NGO respondents are influential. Engineered residential construction remains to be of importance in reducing mortality in developing countries. It is essential that hospitals, fire departments and police stations, water, telephone and electrical facilities be made earthquake proof.

## 1. Introduction

Earthquakes are calamities managed differently in different countries and settings; management depends upon the resources available and the relief feasible. A study to review and compare various aspects of recent earthquakes to assess various issues facing the responders and survivors seems prudent. The medical and nonmedical needs of residents in the disaster areas and the implementation of effective disaster medical aid and task force are essential to decrease the death toll. Evaluation of overall physical and mental health status after earthquakes is important in survivors. Assessment of management and aftermath may shed light on ways to reduce mass casualties in future disasters.

## 2. Materials and Methods

A retrospective literature review of the various aspects of management and aftermath of 5 major earthquakes (in Bam Iran, Sichuan China, Port-au-Prince Haiti, Kashmir Pakistan and Ica Peru) of the past decade (2000–2010) was undertaken. A literature review was done via computer search of published articles (indexed in Pubmed), and available literature to assess: 1)Local magnitude,2) Type of structure 3)Time of earthquake (day/time/season), 4) Search and rescue, 5)Triage, Transfer, and Treatment 6) Casualties (dead/ injured), 7) City damage, 8) Damage to health facilities, 9)Field hospital availability, 10)International aid, 11)Air transfer, 12) Telecommunication systems availability, 13) PTSD prevalence, 14) Most common injury and 15) Disease outbreak.

## 3. Results

### 3.1. Local Magnitude

**Figure 1.**
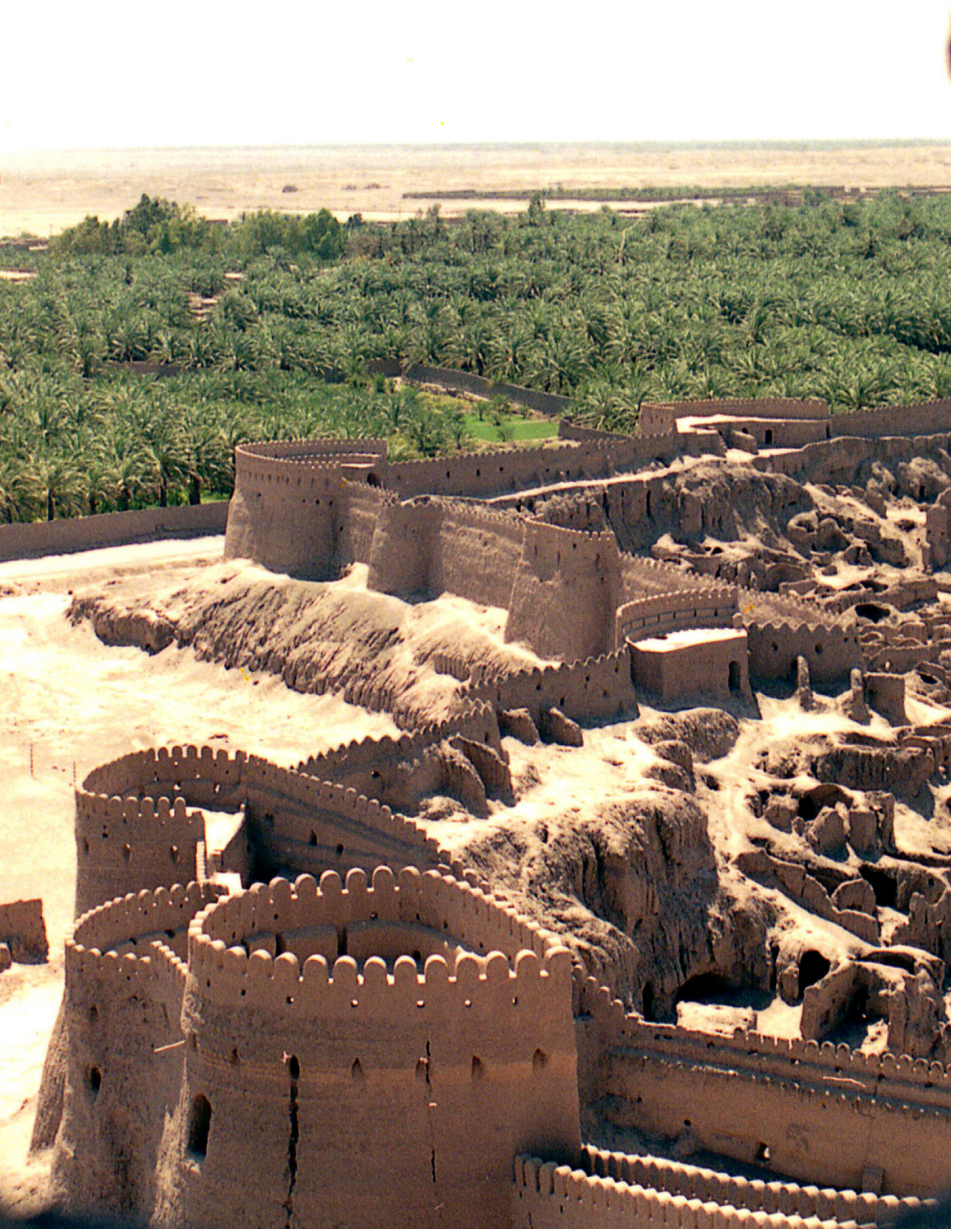
The once magnificent cultural heritage landmark the Bam citadel nestled adjacent to a date palm plantation.

**Figure 2.**
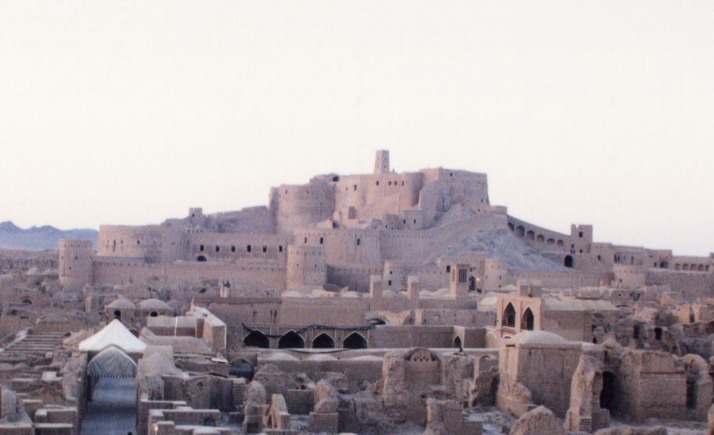
Another view before the quake

**Figure 3.**
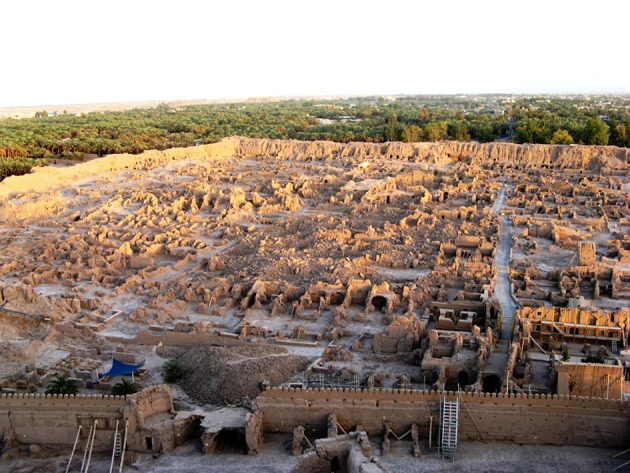
The destruction after the earthquake shows extensive collapse of clay construction.

**Iran.** Bam in southeastern Iran was devastated by an earthquake measuring 6.3-6.6 on the Richter scale early Friday morning on December 26, 2003; it had the lowest magnitude of the 5 (Figures. 1-3)(1-4).

**Figure 4.**
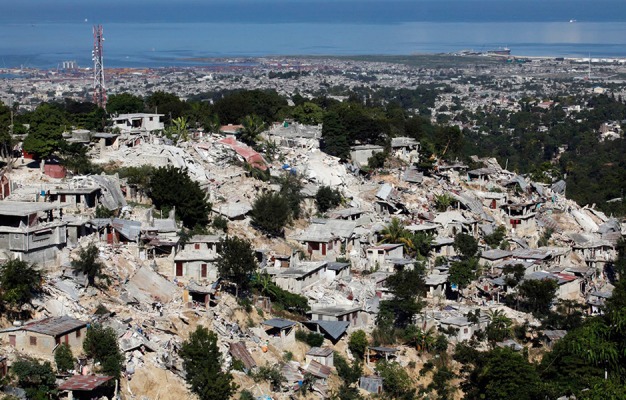
The dire situation in Haiti after the earthquake. Note extensive collapse of nonengineered buildings mimicking a landslide because the buildings are mostly instable when on a slope.

**Figure 5.**
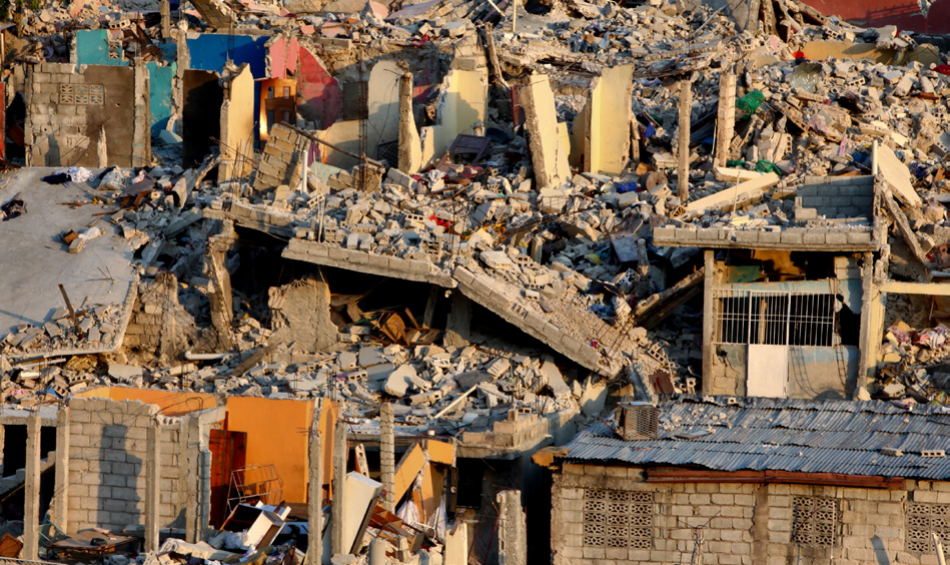
Closer view reveals the crumbling of masonary construction and weak column joints. Note no collapse of nonengineered building with light aluminum roofing on the right.

**Haiti**. Haiti was hit on the evening of January 12, 2010, by an earthquake with a magnitude of 7.2 on the Richter scale (Figures. 4, 5)(5).

**Figure 6.**
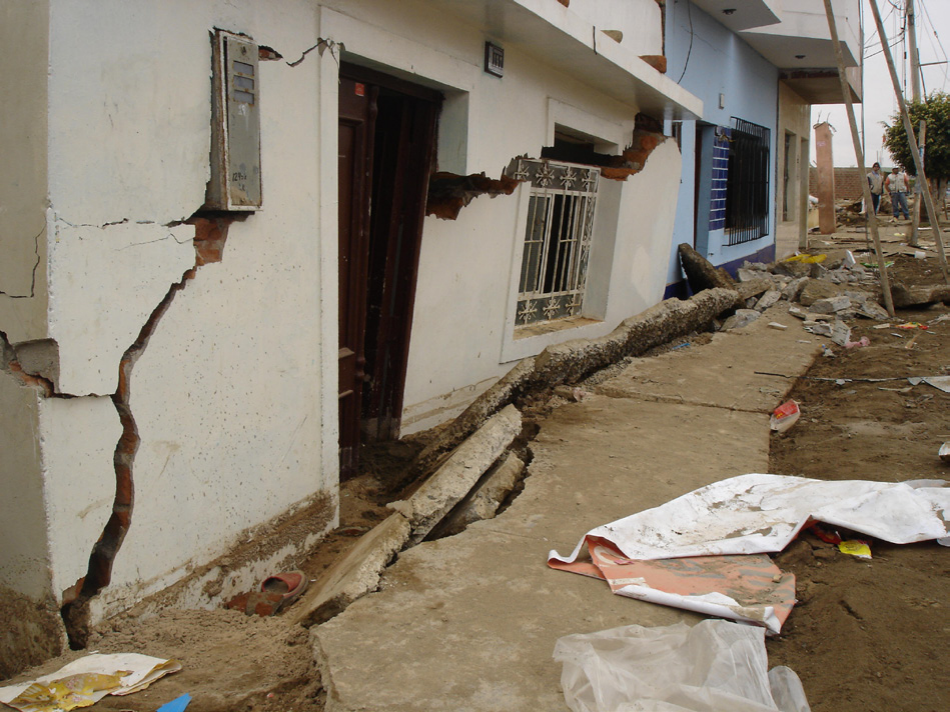
The situation was better in Peru were reinforced concrete was used.However note that 45 degree earthquake cracks from shear forces can be seen on masonary structures.

**Figure 7.**
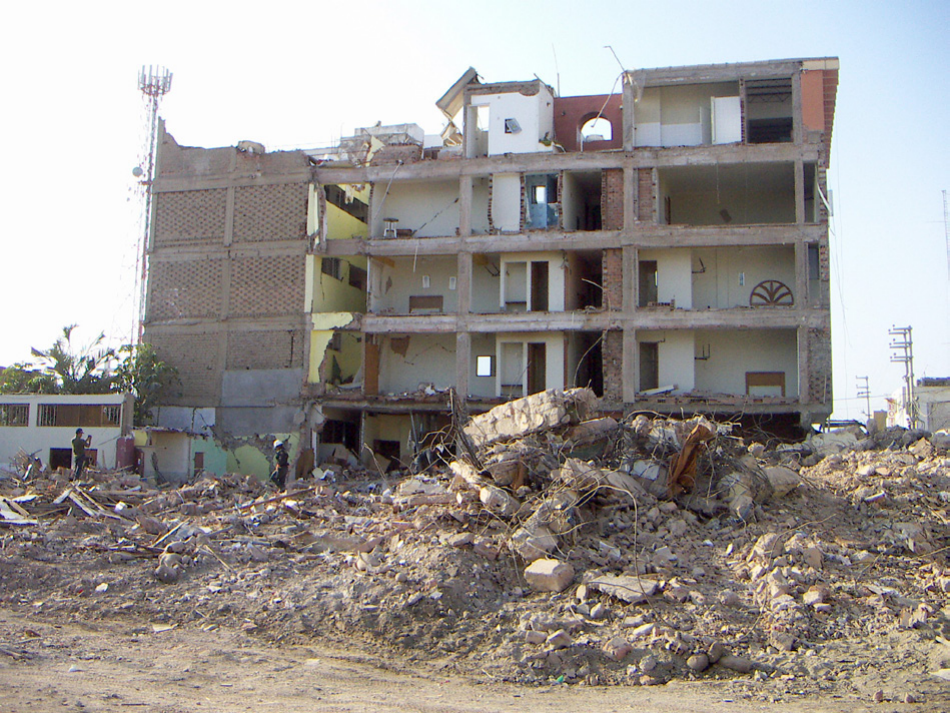
Brick walls collapsed while the infrastructure held up because the concrete structure was sturdy and well designed. However in long spans (on the right),because of poor wall retention all the bricks collapsed.

**Peru**. Peru was hit on August 15, 2007, by a 7.9 Richter earthquake off the western coast of Peru that devastated Ica (Figures.6,7)(6).

**Figure 8.**
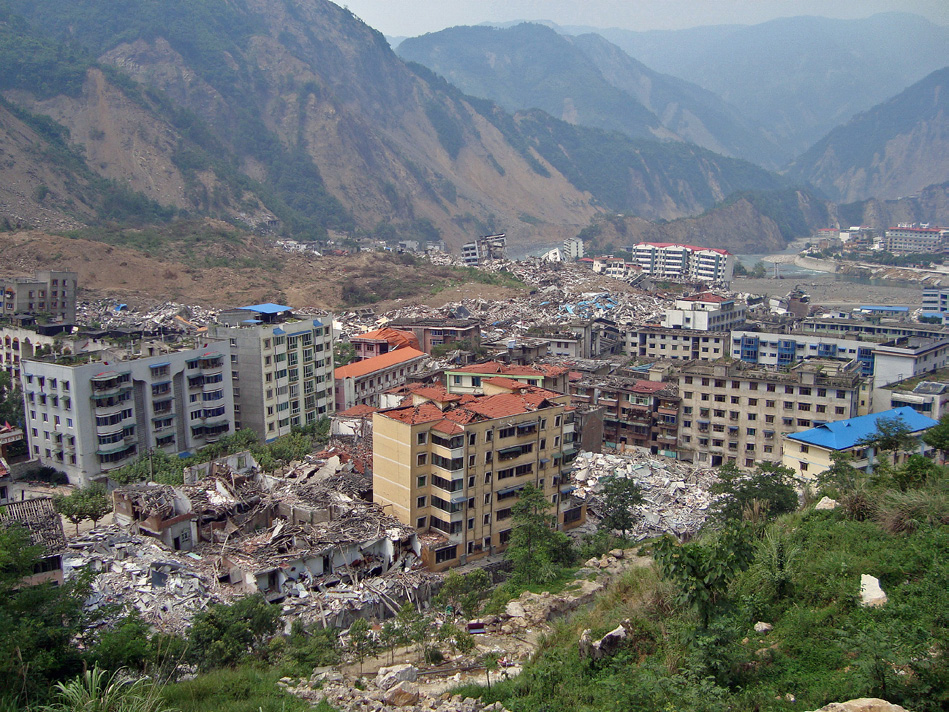
The destruction after the earthquake in Sichuan valley. Note the newer more recently constructed engineered buildings held-up whilst the older poorly engineered ones crumbled. Closer view reveals the crumbling of wood and brick construction but no damage to engineered structure in background.Note building in the center,uprooted from it’s foundation and toppled.

**Figure 9.**
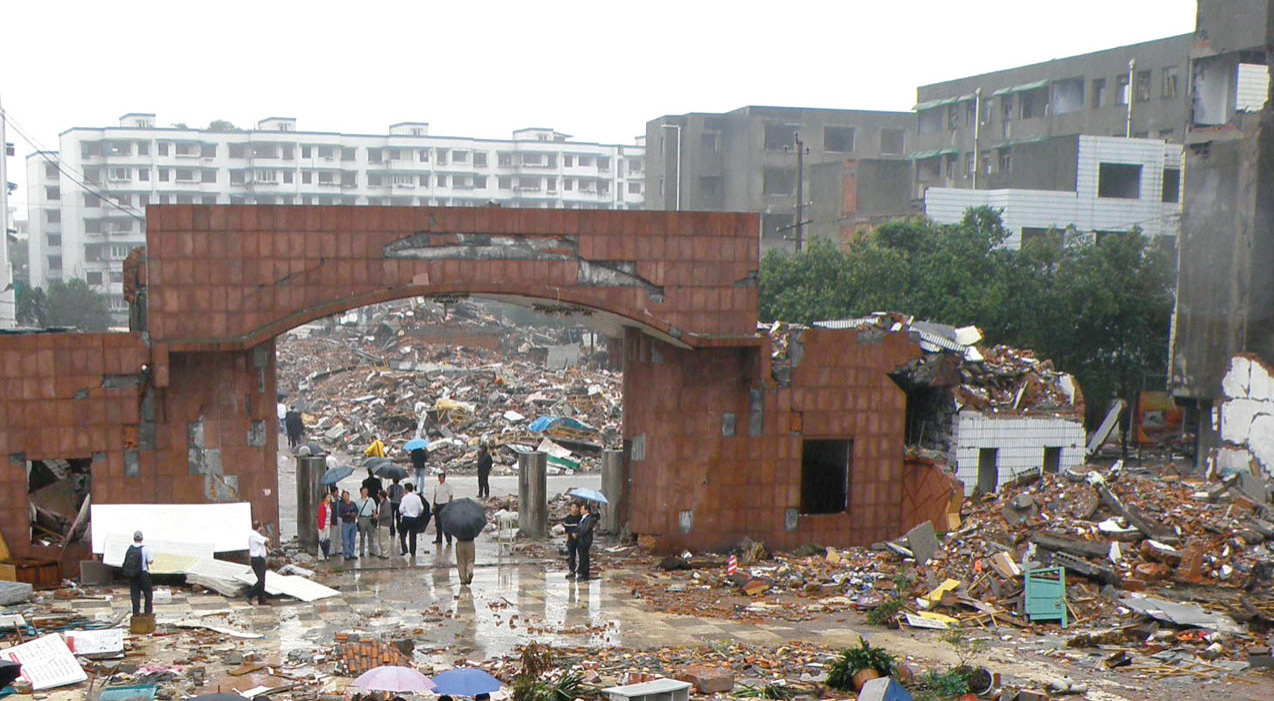
Note again no collapse of more-recent engineered structures and rubble of brick buildings.

**China**. The 8.0 magnitude earthquake that struck Sichuan on May 12, 2008, was the deadliest earthquake in 30 years (Figures. 8-9) and had the greatest magnitude of the 5 (7).

**Figure 10.**
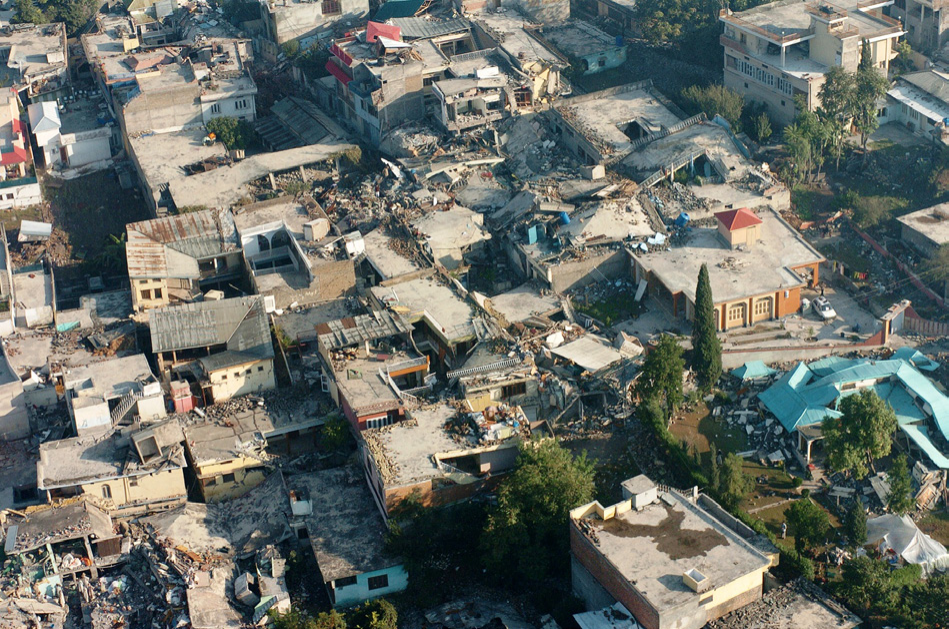
The destruction after the earthquake in Pakistan shows extensively collapsed rooftops due to weak construction of supporting joints.

**Pakistan**. The earthquake that hit northern Pakistan, on October 8, 2005 measured 7.6 on the Richter scale and caused massive destruction (Figure.10) (8).

### 3.2. Type of Building Structure

**Iran**. In Bam, most of the houses were old and made of mud bricks and were completely destroyed. Traditional houses of poor residents in the outskirts of Bam were of clay with heavy clay roofing which proved deadly upon collapse (Figures 3-5) (4).

**Haiti**. Structures in Haiti were stacked bricks, cement blocks or concrete (5).

**Peru**. In Peru structures were reinforced concrete with brick walls that collapsed while the infrastructure held up (Figures 9, 10) (6).

**China**. In Sichuan the newer recently constructed engineered buildings held-up while the older nonegineered ones crumbled (Figures 7-8) (7).

**Pakistan**. In Pakistan the structures were concrete, slab roof type which failed (8).

### 3.3. Time of the Earthquake (Day of the week/time/season) 

**Iran**. Friday (4:53Am) Winter (4).

**Haiti**. Friday (05:26Am) Winter (5).

**Peru**. Monday (02:28 Pm) Spring (6).

**China**. Wednesday (06:40Pm) Summer (7).

**Pakistan**. Saturday (8:52 Am) Autumn (8).

### 3.4. Time to Search and Rescue

**Iran**. In Bam, the mean time under rubble was 4.8±4.9 hours, 16% being trapped for one hour. The average extrication to intravenous infusion was 18.9 hours; a considerable number (23%) of victims received their first intravenous infusion within 12 hours after the quake (earliest:10 minutes, latest 96 hours) (9). In Bam the earthquake was publicly announced 6 hours after its occurrence. Local and international rescue teams reached the scene at the earliest 12 hours after the disaster. (10). A cross-sectional study of 185 casualties who were transferred and admitted to the Bam University Hospital during the first week following the earthquake showed that the mean time taken for the first rescuers to reach the scene and rescue the victims from beneath the rubble was 1.7 ± 2.7 and 0.9 ± 1.1 hours, respectively; In a tertiary referral trauma management center in Tehran, 210 victims were assessed and the average time under rubble was 1.9 hours. The mean time between rescue and final medical treatment was 13.5 hours (9, 10).

**Haiti**. In Haiti, time to rescue and treatment was about 20 hours (5).

**Peru**. Rescue and treatment was rendered on the same day of the earthquake in Peru.

**China**. The first medical team arrived 10 hours after the earthquake from Mianyang City (3, 7).

**Pakistan**. In Pakistan, rescue and treatment was rendered on the same day of the earthquake (8).

### 3.5. Triage, Transfer, and Treatment

**Iran**. At the Bam airport, triage was done during the evening following the tragedy because the field hospitals were not established until the next day, and the two major hospitals in Bam were destroyed. Triage was done primarily in the lobby of the airport, which was full of victims. Once the area was cleared, triage utilized the START (Simple Triage and Rapid Treatment) method categorized the victims into four classes (immediate, delayed, out-patient, deceased). The triage was effective and expedient. Nearly all of the casualties were evacuated from Bam within 48 hours; the Red Crescent Society was responsible for transportation of the victims to the collection point and EMS was responsible for transportation to the advanced medical posts; 68.9% of the patients were admitted to hospitals during a 15 day period after the earthquake (11).

**Haiti**. Triage of casualties in Haiti was done by one medical doctor in a hotel parking lot until arrival of international aid and by an internist or a pediatrician; then an appropriate surgeon (trauma, orthopedic, or neurosurgeon) developed a plan of care (1).

**Peru**. Triage of casualties was not elaborated.

**China**. Triage of casualties was done by grouping (7).

**Pakistan**. Triage of casualties was done by 2 general doctors and 2 paramedics at the entrance to the hospital (8).

### 3.6. Casualties and Distribution of Dead/ Injured

**Iran**. The Statistical Center of Iran reported 26,271 killed, based on the identification documents rendered void (1-4). In Bam there were 26,271 dead, 51, 500 injured and nearly 12,000 patients transferred to hospitals (9, 10). The worst dead to injured ratio was 51% in Bam (9-11).

**Haiti**. The southwest of Haiti, including the capital Port-au-Prince, provoked immense human and material damages due to the with 212,000 dead, 512,000 wounded and 1,000,000 homeless (5). Haiti suffered the deadliest earthquake of the past decade but the dead to injured ratio was better than Bam at 42%.

**Peru**. Peru had 514 people killed and 1090 injured (6). In Peru, mortality to injury rates in the four most affected provinces were estimated at 1.4 deaths/ 1,000 exposed (95 CI: 0.5-3.3) and 29 injuries/1,000 exposed (95 CI: 6-52). Older adults and members of households of lower socioeconomic status had increased injury. No significant differences in injury rates were observed between rural, urban, and suburban residential areas (12). Dead to injured ratio was 32%.

**China**. The State Council of China pronounced 69,170 people dead, 17,426 missing, and 374,159 injured (7). This earthquake had the best dead to injured ratio which was 15 % .

**Pakistan**. The Pakistan earthquakes’ official death toll was 73,276 and over 80,000 severely injured. It had the next to the worst dead to injured ratio which was 47% (8).

### 3.7. Degree of City Damage

**Iran**. The damage was extensive, and most buildings (85%) were destroyed within the impact zone. Approximately 18,000 buildings were destroyed and some 75,000 left homeless (1-4).

**Haiti**. Extreme damage was seen; the deputy mayor of Léogâne reported that 90% of the town's buildings had been destroyed (5).

**Peru**. More than 35,500 buildings destroyed and more than 4,200 buildings damaged (6). The number killed was 514.

**China**. About 80% of the buildings were destroyed.

**Pakistan**. About 60% of the buildings were destroyed in Muzaffarabad or had collapsed, and an estimated 3.3 million people were rendered homeless (8).

### 3.8. Degree of Damage to Health Facilities 

**Iran**. Bam and Pakistan earthquakes had the highest health facility damage (90%). The Bam earthquake in Iran reportedly destroyed almost all of the health facilities in the affected area, with the loss of almost 50% of the local health staff; all of the hospital facilities were destroyed and all the physicians and nurses were injured or killed; large numbers of injured people were evacuated to hospitals throughout Iran; the collapse of health facilities in Bam was enormous. On the day of the earthquake, only first aid and limited necessities for basic prehospital and advanced trauma life-support were provided. Nearly all health service facilities and 120 schools were destroyed. Both hospitals in Bam were destroyed; although both were supposedly engineered neither proved earthquake proof (13).

**Haiti**. The earthquake that struck Haiti damaged 22%of the hospitals in the entire country. The initial hospital was a small facility within the United Nations compound that rapidly outgrew itself. The second hospital was constructed 4 days later (14). In Haiti damage to buildings and essential services such as power and water supplies, hospitals and government services was extensive, in part because of poor building standards and already overstretched services.

**Peru**. The healthcare infrastructure was devastated. After the earthquake 60% of health facilities in the affected area reported some damage and 4 were destroyed, but 80% continued to provide services after the event (6).

**China**. Most hospitals were destroyed and limited facilities were available for medical service in the earthquake regions. In Beichuan County, 80% of the buildings collapsed (7, 15).

**Pakistan**. About 90 percent of health facilities in the affected area suffered damage (16).

### 3.9. Field Hospital Availability

**Iran**. After establishing field hospitals (48 hours after the earthquake), more advanced immediate medical and surgical care was rendered (9, 11, 17).

**Haiti**. The first field hospital was available after 24 hours. In Haiti the Argentine military field hospital, which had been serving Minustah, was the only one available until 13 January, 2010 (18).

**Peru**. Several countries provided field hospital aid.

**China**. The first field hospital was available after 24 hours.

**Pakistan**. The first field hospital was available after 72 hours. In Pakistan the International Committee of the Red Cross (ICRC) sent a field hospital to Muzaffarabad on 11 October 2005.;28 days after the earthquake 16 field hospitals and 44 basic healthcare units had been registered in the area (19). In Pakistan no civil administration remained; water and sanitation were in a deplorable state, minimal power or communications had been restored, and 90% of health care infrastructure was ruined, including the main District Hospital (16).

### 3.10. International Aid

**Iran**. In Bam more than 40 international teams provided search and rescue services and aid. There also were many volunteer aid groups at the scene (11).

**Haiti**. In Haiti over 20 countries provided aid (14).

**Peru**. Several countries provided international aid.

**China**. In China more than 130,000 troops were dispatched to the affected areas; 19 international teams provided aid (15).

**Pakistan**. A significant number of countries provided international aid (19, 20).

### 3.11. Air Transfer

**Iran**. The first plane carrying specialized teams landed in Bam almost 14 hours after the earthquake. The total number of injured transferred by air 9 days after the incident was 5,511; 54 percent of injured were evacuated by air (3, 10).

**Haiti**. In Haiti 600 emergency flights had landed within 5 days (14).

**Peru**. Air transfer was available to evacuate the injured from the earthquake site.

**China**. Air transfer was available to evacuate the injured from the earthquake site.

**Pakistan**. In Pakistan the injured required United Nations or military helicopters because landslides and aftershocks had made the mountainous roads impassable; and in 4 weeks after the earthquake, it was estimated that a total of 20,000 patients had been transferred by helicopter to cities outside the affected area. In Pakistan the roads were destroyed and initially the only way to transport and transfer the injured was by air (16, 19, 20).

### 3.12. Telecommunication Systems Available

**Iran**. Because the mass media communication channels (including cell phone, fax and email) in Bam were destroyed the survivors were unable to seek rescue aid for hours (21).

**Haiti**. In Haiti because of the massive damage to the communications infrastructure, satellite links for telephone and internet connections were used. The command groups in Haiti and Miami had joint conference calls every morning, during which the day’s goals and plans were set. In Haiti the Dominican Institute of Telecommunications (Indotel) helped with the restoration of some telephone services and the Télécoms Sans Frontières (TSF) had opened a BGAN connection and a wireless access point for UN agencies and NGOs. Due to the continuous growth of rescue teams coming to Haiti, TSF teams opened a VSAT connection for OCHA’s (UN Office for the Coordination of Humanitarian Affairs) and ECHO ‘s (European Commission’s Humanitarian Aid Department) teams, also used by other international humanitarian organizations (14, 18).

**Peru**. In Peru the relief team from TSF’s American base, composed of 5 specialists in emergency communications transports satellite communication equipment (Inmarsat BGan terminals, RBGan, GAN M4 and Mini M) were used as well as computer equipment to provide internet connections, telephone lines and fax (22).

**China**. China CDC developed a reporting system based on the short messaging system (known as SMS or text messaging) which was effective (23, 24).

**Pakistan**. In Pakistan no communication or rescue service was available during the first 3 days (25).

### 3.13. PTSD Prevalence

**Iran**. About 58% of 961 survivors in Bam assessed in 2004 and 81% of the 145 participants in 2003 had developed PTSD according to Diagnostic and Statistical Manual of Mental Disorders (DSM-IV or DSM IV-TR) criteria (26). A crosss-ectional study of 400 persons selected by stratified, multi-stage area sampling of survivors interviewed in temporary residential camps showed complicated grief was detected in 304 (76%) of the respondents (27).

**Haiti**. Depression, anxiety and grief were prevalent in survivors. 

**Peru**. PTSD prevalence in Peru was 25.2%. In a bivariate analysis, PTSD was significantly associated with the female sex, loss of church, food and water shortages immediately after the earthquake, joblessness, injuries, loss of a relative or friend, lack of clean drinking water or inappropriate sleeping conditions, 5 months after the earthquake, and low levels of perceived support from family and friends. In the multivariate analysis, only female sex, food and water shortages, loss of church, injuries, and low levels of perceived support from family and friends were independently associated with PTSD (28).

**China**. Prevalence of PTSD was 45.5% (203/446). The point prevalence rates of posttraumatic stress disorder (PTSD) and depression among the child survivors aged between 8 and 16 years, 15 months after the earthquake were 12.4 and 13.9%, respectively. Children who had lost family members developed PTSD as well as depression. Children who reported no utilization of mental health services were four times more likely to suffer from PTSD than those who did not (28-31). 62.8% of subjects met the criteria for diagnosis of PTSD 1 month after the earthquake (32). Another study in China showed low household income, being from an ethnic minority, living in a shelter or temporary house, death in the family, and household damage were factors significantly related to increased odds of PTSD (29).

**Pakistan**. A substantial subgroup of participants reported clinically relevant levels of emotional disorders, especially earthquake-related posttraumatic stress disorder (42.6%), as well as depression and anxiety (approx. 20%). Symptom levels of PTSD were associated with the severity of the earthquake experience, previous trauma, work-related stressors, low social support, and female gender (33).

### 3.14. Most Common Injury

**Iran**. In Bam extremity fractures (19%) were more common than were axial skeleton fractures (4%) (21).

**Haiti**. In Haiti, the most common injuries were injuries of the knee, lower leg, ankle and foot (36%), followed by head injuries (18%). In the children’s hospital, the most frequently seen injuries were also of the knee, lower leg, ankle or foot (19%), and of the abdomen, lower back, lumbar spine, pelvis, and thigh (15%) (34).

**Peru**. Extremity fractures were more common.

**China**. In China blunt trauma, crush injury and falls were common seismic injuries. The most frequently injured site was the lower extremity. The main injury was fracture of the lower extremities, pelvis and the spine. The lower extremities had the highest incidence of injury, accounting for up to 39.0% (35, 36).

**Pakistan**. In Pakistan 78 patients with a mean age of 23 years were treated; Fifty-two patients had only lower limb injuries (66%), 18 upper limb injuries, and 8 had combined lower and upper limb trauma (37). Another report in Pakistan states the most common bone injury was lower limb fracture (52%) (20).

### 3.15. Most Common Disease Outbreak

**Iran**. Acute upper-respiratory-tract infection was a major problem in Bam (21).

**Haiti**. In Haiti: the three most frequently reported specified conditions were acute respiratory infection (16.3%), suspected malaria (10.3%), and fever of unknown cause (10.0%) (38).

**Peru**. In Peru, surveillance system for infectious diseases confirmed that respiratory infections (bronchitis, pneumonia, etc.) were frequent (39).

**China**. In China after the seventh day, acute upper-respiratory-tract infection was the leading problem (40).

**Pakistan**. In Pakistan 2, 194 patients treated by the Chinese Medical Rescue Team, stated trauma patients only accounted for 29%, diarrhea patients for 4%, upper respiratory infection patients for 14%, and other types of diseases for 52% (41). During a 3-week period , 316 patients were treated at the hospital; 246 were women and children (77.9%). The majority of patients had infected wounds with or without fractures (42). Acute respiratory infections (ARIs) were the most frequent causes of concern after the earthquake (19).

## 4. Discussion

During the past decade 5 major earthquakes (excluding tsunamis) occurred; Haiti was the deadliest. The pattern of injuries during the earthquake showed a higher proportion of females and lower limb bone injuries. Lower extremity injuries were predominant in all states similar with that in the Indian Ocean and the Marmara earthquakes. The data highlights the need to address orthopedic, pediatric, and women’s health issues as well as respiratory infections that were common in all 5 countries.

As deaths are due to collapsing structures thus this issue warrants greater attention.

### 4.1. Type of Buildings and Quality of Construction

“Where there are no buildings, there is no damage...’’ A building built without strict specifications to withstand earthquakes of high magnitudes will suffer extensive damage even during a moderate earthquake (36). In Haiti the reason for the disaster is clear in the mangled ruins (Figures 4, 5) the buildings had been doomed during their construction (19); the best action plan to reduce damage and injuries in medicine is via preventive measures. Since we cannot prevent earthquakes, a way must be found to reduce injuries and minimize damage. This can be achieved by implementing strict building standards according to the type of building. The effectiveness of regulatory measures was proven during the 1989 San Francisco earthquake. All hospitals built after the new standards were implemented withstood the quake and continued functioning. Some older hospitals, built prior to the introduction of the new laws, were damaged (36). Collapse of rooftops was also seen in Pakistan (Figure. 10). The greater the magnitude of the earthquake does not necessarily mean a higher death toll (Tables 1 and 2); Haiti had more deaths than China despite lower quake magnitude. 

**Table 1.** Comparative data relevant to major earthquakes of the past decade.

	Date	Local magnitude (Richters)	Type of constructions damaged	Time of the event(day/ time/ season)	Time to rescue and treatment	Ratio of casualties: dead/ injured (%)	City Damage, %	Health facilities damage, %
Iran (Bam)	2003 Dec	6.6	Clay bricks, Masonary	Friday (4:53 Am) Winter	12 hours after	26,271/51,500: (51)	85	90
Haiti (Portau- Prince)	2010 Jan	7.2	Stacked bricks, cement blocks or concrete	Friday (05:26 Am) Winter	20 hours after	212,000/512,000: (42)	90	22
Peru (Ica)	2007 Aug	7.9	Concrete and brick	Monday (02:28 Pm) Spring	The same day	514/1,604: (32)	Most of the city	60
China (Sichuan)	2008 May	8.0	Brick and Concrete	Wednesday (06:40 Pm) Summer	The same day	69,170/443,329: (15)	80	N/A
Pakistan (Kashmir)	2005 Oct	7.6	Concrete, slab roof	Saturday (8:52 Am) Fall	The same day	73,276/ 153,276:(47)	60	90

**Table 2.** Comparative data relevant to major earthquakes of the past decade.

	First Field Hospital	Intnational Aid	Air Transfer	Mortality	Type of field Triage	Tele Com systems used to summon aid	PTSD prevalence, %	Most Common injury	Most Common disease
Iran (Bam)	After 48 hours	40 countries	5,521 / 9 days	25,000	START and SAVE	None for hours Satellite phone & cell phone	58	Lower ext	Respiratory infection
Haiti (Port-au-rince)	After 24hours	Over 20 countries	600 EMS flights /5 days	212,000	Triage by one medical doctor in a hotel parking lot until arrival of international aid	Satellite phone & wireless access point ( provided by TSF)	Depression, anxiety, grief was prevalent	Lower ext	Respiratory infection
Peru (Ica)	Available	Several countries	Available	514	N/A	Internet connections, telephone lines and fax ( provided by TSF)	25.2	Lower ext	Respiratory infection
China (Sichuan)	After 24 hours	About 20 countries	Available	73,276	Grouping	Mostly SMS and internet online center	45	Lower ext	Respiratory infection
Pakistan (Kashmir)	After 72hours	Significant number of countries	20,000 /4 weeks	69,170	By 2 general doctors and 2 paramedics at the entrance to the hospital	None for 3 days Satellite phones ( provided by TSF)	42.6	Lower ext	Wound inf., respiratory inf.

### 4.2. Time of the Earthquake

An earthquake that occurs in the middle of the night when people are sleeping may cause a greater number of injuries than at midday. Another component affecting the rate of injury, morbidity and mortality is the day of the week and the season of the year (i.e. victims may freeze in the winter) (36). The time of the event is an important variable because during early hours of the day and on weekends people are indoors and this factor can increase mortality and morbidity. This factor was significant in Bam and Pakistan when the earthquake hit. In Bam the quake happened during the very early hours of the day (when most were asleep) and on Friday (nonworking day in Iran) when most people were indoors spending the weekend at home; this further contributed to morbidity and mortality. In Pakistan the quake hit in the month of Ramadan (when people fast); most people were taking a nap after their pre-dawn meal and did not have time to escape during the earthquake. 

### 4.3. Telecommunication Systems

The literature shows that the telecommunication system collapses during a quake and it is impossible to call for help. Cell phone service is also unreliable in an emergency and only radio communication systems function (36). Because the mass media communication channels (including cell phone, fax and email) in Bam were destroyed the survivors were unable to contact and seek aid for hours (21). In Pakistan no communication or rescue service was available for 3 days (36).

### 4.4. Search and Rescue

Survival is directly related to the length of time from the occurrence of the quake until the time of extrication from under the rubble. The 1990 earthquake in the Philippines, showed that 99% of all victims who were extricated alive during the first 48 hours survived (36). The incidence of compartment syndrome had a direct relation to the time under rubble, and the incidence of renal failure was directly related to the rescue-to-first medical aid time (43). Schultz et al. compared three earthquakes in populated areas: the 1976 earthquake in China, the 1980 quake in Italy, and the 1988 quake in Armenia. They found that 85-95% of all surviving extricated casualties had been pulled out from the rubble within 24 hours. They also found that in the earthquakes of Turkey and China, victims trapped for 2 to 6 days under the rubble had only a 50% survival rate. The authors quote Safar who stated that 25 ± 50% of all earthquake fatalities during the Italian earthquake could have been saved had they received medical treatment immediately (36). A database study of 2,086 victims that were hospitalized within the first 10 days following the earthquake found that a longer time under the rubble was associated with acute renal failure and late hospitalizations (43, 44). Soon after the quake, China’s Health Ministry said that it had sent 10 emergency medical teams to Wenchuan County in southwest China’s Sichuan province on the same day. In Bam, one of the major shortcomings was insufficiency of equipment and dogs, which prevented digging in the proper place in search of surviving victims under the rubble; now trained dogs have been purchased by RCS for this purpose. To overcome this long time-delay, rescue teams have now been established in all cities (by the Red Crescent, national universities and Basij task forces) (3). Medical response was delayed as well in Beichuan and according to You et al. response time is very important as many injured people urgently need medical care. The family and neighbors play a major role in helping find and evacuate the injured. 

### 4.5. Triage Issues and Field Hospital Availability

Hospital availability during earthquake disasters is of paramount importance. The ability of the hospital to continue functioning depends on the magnitude of the quake and the construction standards applied (36). On-scene compared with hospital triage methods START (Simple Triage and Rapid Treatment) and SAVE are the widely used triage procedure models for primary and secondary triage of victims at a disaster scene; Many researchers debate triage methods and their accuracy, and there is no existing ‘‘gold standard’’ in either the trauma or disaster literature against which to judge the accuracy or appropriateness of mass casualty triage decisions (11); some institutions dispatched highly specialized surgeons, physicians, cardiovascular experts, ophthalmic experts, or urological experts to offer help. Many of these experts could not function effectively in the field, either because local people had no such needs or because they lacked access to the specialized equipment they needed for their work (36).

### 4.6. International Aid

In Bam although international NGOs did their best to help people in the earthquake region, they also had some adverse impacts on the community in the disaster affected areas. The problems originated from lack of knowledge of cultural issues, inefficient timing for the delivery of funds and services, uneven distribution of goods, and poor communication with local people and authorities (17). Pakistan’s earthquake provoked a significant internationally coordinated response that focused on ensuring the survival of an affected population (43). International aid was significant among the earthquake –hit states. In Bam at first, the true extent of the disaster was under-estimated, but soon became apparent. During the first 6 days following the quake, foreign aid arrived and helped in the relief phase and also established a number of field hospitals.

Approximately 40 countries provided foreign aid. Arrival was late because of late notification (approximately 13 hours after the earthquake). Their teams had between 7 and 80 personnel, between 4 and 22 doctors, between 5 and 200 beds, and performed between 2 and 483 operations during their stays. Since all victims were evacuated within the first 2 days, there were no patients with major surgical needs upon the arrival of foreign aid. Thus, in order to be effective, these teams must be notified early and arrive early after an earthquake (21). The real first responders in the first 24 hours are always the bystanders, who save more lives than the professionals (23). Planes and helicopters play an important role because landslides and aftershocks make the roads impassable (36). Earthquakes severely damage roads and highways and thus, impede the transportation of aid forces, equipment, and supplies (3).

### 4.7. Ratio of Dead to Injured

One of the crucial considerations is the number of fatalities among the total number of injured. A University of Massachusetts study examined this ratio in various earthquakes with a Richter scale magnitude of between 6.5 and 7.4. The ratio was found to be 1:3 dead to injured. In the 1976 earthquake in Nicaragua 22,778 of 76,504 casualties died. During the 1995 Kobe earthquake, only' 527 fatalities were counted among 6,107 casualties, resulting in an 8.6% fatality rate. During the 1988 earthquake in Armenia many towns and villages were devastated. The fatality rate was 80.9% in the town of Spetak, which was completely destroyed (36); this ratio shows both the severity of the earthquake as well as the effectiveness of management as both can effect morbidity and mortality. 

### 4.8. Major Medical Problems

Mental disorders, heart problems, respiratory diseases and many other medical conditions are aggravated by earthquakes. Infectious diseases and outbreaks of epidemics in conjunction with the earthquake also affect the health and welfare of the public; psychological stress among the medical teams is another important issue (35, 39, 45). Psychological distress measured in Bam using the 12-item General Health Questionnaire (GHQ-12) in 916 survivors showed that 58% of the respondents suffered from severe mental health problems (three times higher than the prevalence of psychological distress among the general population.) The results of the logistic regression analysis indicated that female gender, lower education, unemployment, and loss of family members were causes linked to severe psychological distress among earthquake victims (9).

Post-Traumatic Stress Disorder or PTSD among survivors had the lowest prevalence in Peru and highest prevalence in Bam. Psychological counseling was needed for those who survived the event (25). The finding of complicated grief required more attention of mental health services. Rebuilding of homes, finding a place for survivors to live, and respecting cultural and ethnic customs of the survivors are all factors in helping survivors to regain their mental health within a shorter period of time (27). A higher incidence of abnormal menstruation may occur in students with PTSD, somatization disorder, obsessive-compulsive disorder, phobic anxiety, poor diet and sleep disorders. Therefore, psychological intervention is particularly necessary for female students who have survived a natural disaster like an earthquake (30). Married status, female gender, having death or injured family members, low educational level, and loss of possessions all had significant effects on survivors with PTSD (32).

## 5. Conclusions

Responses to and relief efforts in an earthquake zone are dynamic and change rapidly. Numerous factors are influential. Field hospitals must be prepared for adjustment to their mode of activity and for extreme conditions. Injury and fractures were main problems among the patients following an earthquake. The key point in the management of supplies and equipment during a disaster is to provide what is needed at the right time, and a planned program to cope with the needs and requirements of the situation is what guarantees effective performance of a treatment center in a disaster; improvement of healthcare facilities, and provision of organized communication channels between the different governmental departments are basic tools for operating a proper command system and instituting interagency coordination among relief workers. Continuous education, training the general population and the people involved in disaster management is paramount. Postdisaster mental health recovery programs that include early identification, ongoing monitoring, preventive and intervention programs, and sustained psychosocial support are needed for the highest-risk population, namely, the bereaved, people without income and those with serious household damage.

By reviewing the data from past disasters it is apparent that contemporary earthquake management should include the following: 1. Timely activation of rescue teams; 2. Training rescue teams according to a standardized curriculum; 3. Establishing emergency medical care at the scene in the first hours after earthquake; 4. Providing prompt air transfer services for the critically ill victims; 5. Applying the quick assessment and management charts/ forms at the scene by paramedics; 6. Establishing a national on-line data bank to collect and analyze the data; 7. Building quake-resistant structures; 8. Continuous training programs for students in schools and universities as well as the general population; 9. Permanent disaster relief team organizations with national and international activities; 10. Developing strategic managing plans; 11. More investment to make communities better prepared for disaster and less vulnerable; 12. Volunteer aid recruiting (and educating to prevent harm to victims upon rescue) and training the general public to deal with major emergencies; 13. Social organizations should be well organized to participate in assistance; 14. All treatment workers should be dressed uniformly; 15. Ideally, disaster management should be an essential component of medical training.

It is of note that in this review the lack of healthcare workers to triage and treat the wounded was insufficient. Earthquakes occurring at night carried a higher death toll. Engineered residential construction remains to be of utmost importance in reducing mortality. Thus, it is imperative that hospitals, fire department and police stations, etc. be made earthquake-proof.
